# Reassessment of areas with persistent Lymphatic Filariasis nine years after cessation of mass drug administration in Sri Lanka

**DOI:** 10.1371/journal.pntd.0006066

**Published:** 2017-10-30

**Authors:** Ramakrishna U. Rao, Sandhya D. Samarasekera, Kumara C. Nagodavithana, Tharanga D. M. Dassanayaka, Manjula W. Punchihewa, Udaya S. B. Ranasinghe, Gary J. Weil

**Affiliations:** 1 Washington University School of Medicine, St. Louis, MO, United States of America; 2 Sri Lankan Ministry of Health, Anti-Filariasis Campaign, Colombo, Sri Lanka; 3 Regional Anti-Filariasis Unit, Galle, Sri Lanka; Imperial College London, Faculty of Medicine, School of Public Health, UNITED KINGDOM

## Abstract

**Background:**

Sri Lanka was one of the first countries to initiate a lymphatic filariasis (LF) elimination program based on WHO guidelines. The Anti-Filariasis Campaign provided 5 annual rounds of mass drug administration (MDA) with diethylcarbamazine plus albendazole in all 8 endemic districts from 2002–2006. Microfilaremia (Mf) prevalences have been consistently <1% in all sentinel and spot-check sites since 2006, and all evaluation units passed school-based transmission assessment surveys (TAS) in 2013. We previously reported results from comprehensive surveillance studies conducted in 2011–2013 that documented low-level persistence of *Wuchereria bancrofti* in 19 high risk areas in 8 endemic districts. We now present results from repeat surveys conducted 3 to 4 years later in 6 areas that had the strongest LF signals in the prior study.

**Methodology and principal findings:**

The surveys assessed prevalence of filarial antigenemia (CFA) and Mf in communities, CFA and anti-filarial antibody in school children (ages 6–8), and filarial DNA in *Culex* mosquitoes (molecular xenomonitoring, MX). Three study areas had significantly improved infection parameters compared to the prior study, but three other areas had little change. MX was more sensitive for detecting *W*. *bancrofti* persistence, and it was a better predictor than other parameters. Adult males accounted for more than 80% of infections detected in the study.

**Conclusions:**

These results suggest that *W*. *bancrofti* transmission was near the break point in some of the areas studied in 2011–13. LF is likely to decline to zero without further intervention in these areas, while other areas may require further intervention. Long term surveillance may be needed to verify *W*. *bancrofti* elimination in areas like Sri Lanka with efficient transmission by *Culex*. Test and treat or other programs targeting adult males plus bed net promotion may be more effective than MDA for clearing remaining hotspots of transmission in Sri Lanka.

## Author summary

Lymphatic Filariasis (LF, also known as “elephantiasis”) is a disabling and deforming tropical disease caused by parasitic worms that are transmitted by mosquitoes. The Sri Lankan Anti-Filariasis Campaign provided 5 annual rounds of mass drug administration (MDA) with diethylcarbamazine and albendazole between 2002 and 2006 in all endemic areas, and this reduced infection prevalence to very low levels. Post-MDA surveillance conducted by our group in 19 sentinel sites in 8 endemic districts in 2011–2013 revealed evidence of persistent LF infection in all study sites. The present paper reports results of repeat assessments conducted 3–4 years later in 6 areas with high signals in the prior study. LF parameters were significantly improved in 3 areas where LF appears to be on a glide path to elimination. However, LF infection parameters remained high in 3 areas, and further work will probably be required to interrupt transmission in these areas. Molecular xenomonitoring (to detect filarial DNA in mosquito vectors) was especially sensitive for detecting persistent LF in Sri Lanka, and this may also be true in other areas with *Culex* transmission. Our results suggest that test and treat or other programs targeting adult males plus expanded bed net use may be helpful for clearing up remaining LF hotspots.

## Introduction

Lymphatic filariasis (LF, caused by the filarial nematodes *Wuchereria bancrofti*, *Brugia malayi*, and *B*. *timori*), is a major public-health problem in many tropical and subtropical countries. The global program to eliminate Lymphatic Filariasis (GPELF) has made significant progress by providing more than 6 billion treatments with antifilarial medications to more than 800 million people in some 60 countries between 2000 and 2015 [1]. This mass drug administration (MDA) program has cured millions of infections and prevented millions of new clinical filariasis cases [1–3]. Countries with successful MDA programs are now looking for additional guidance on stopping MDA and on post-MDA surveillance beyond WHO current guidelines[1, 4, 5] that rely heavily on testing school aged children for filarial antigenemia as a means of demonstrating that transmission of new infections has been interrupted. While such “transmission assessment surveys” (TAS) can be a useful surveillance tool [4, 6], they have not been adequately validated as an indicator for interruption of LF transmission at the evaluation unit or country level. Indeed, prior studies by our group have shown that TAS was not sensitive for detecting ongoing transmission of *W*. *bancrofti* in Sri Lanka [7], and this is likely to be true in many other settings.

Lymphatic filariasis has been endemic in Sri Lanka for hundreds of years [8–11]. The country’s Anti Filariasis Campaign (AFC, established in 1947) implemented control activities over many years that succeeded in reducing infection prevalence to low levels by 1999. After providing mass drug administration of diethylcarbamazine (DEC) for three years starting in 1999, the AFC provided five annual rounds of MDA with diethylcarbamazine (DEC) plus albendazole in all 8 endemic districts (implementation units, IU) between 2002 and 2006 [2, 12–14]. The AFC conducted post-MDA surveillance activities according to WHO guidelines, and all evaluation units in endemic districts easily passed TAS in 2013 [7]. Based on this and other considerations, WHO recognized that Sri Lanka had eliminated LF as a public health problem in 2016, but recommended that the country continue treatment interventions with high population coverage and post-MDA surveillance in isolated foci with evidence of ongoing transmission [1, 15, 16].

We assessed the status of *W*. *bancrofti* in Sri Lanka with comprehensive post-MDA surveillance in 19 Public Health Inspector areas that were considered to be at risk for persistent infection. Comprehensive surveillance comprised community surveys for circulating filarial antigenemia (CFA) and microfilaremia (Mf), school surveys for CFA and antifilarial antibodies, and systematic sampling of *Culex quinquefasciatus* for the presence of filarial DNA (molecular xenomonitoring or MX) [7]. All 19 sentinel areas studied had evidence for persistent *W*. *bancrofti*, but some areas had stronger signals than others. Based on results of that study, we suggested revised endpoint targets for filariasis elimination programs in areas with *Culex* transmission based on upper 95% confidence limits as follows: CFA <2%, antibody prevalence in primary school children <5%, and filarial DNA prevalence in gravid, semigravid, or fed *Culex* mosquitoes <1% [7]. In the present study we have repeated comprehensive surveillance in 6 areas with the strongest signals for *W*. *bancrofti* persistence in 2011–2013 to determine whether there was evidence for improvement or worsening of infection parameters 3 to 4 years after the prior study.

## Methods

### Ethical review and consent procedures

The study protocol was reviewed and approved by institutional review boards at Washington University School of Medicine, University of Kelaniya and at the Ministry of Health in Sri Lanka. Printed copies of participant information sheets (PIS) and written consent forms were provided to participants (or to parents/guardians) in Sinhalese, Tamil and English. Written consent was obtained from adults; participation of minors required written consent from at least one parent or guardian plus assent by the child/minor.

### Study sites

**Fig 1:**
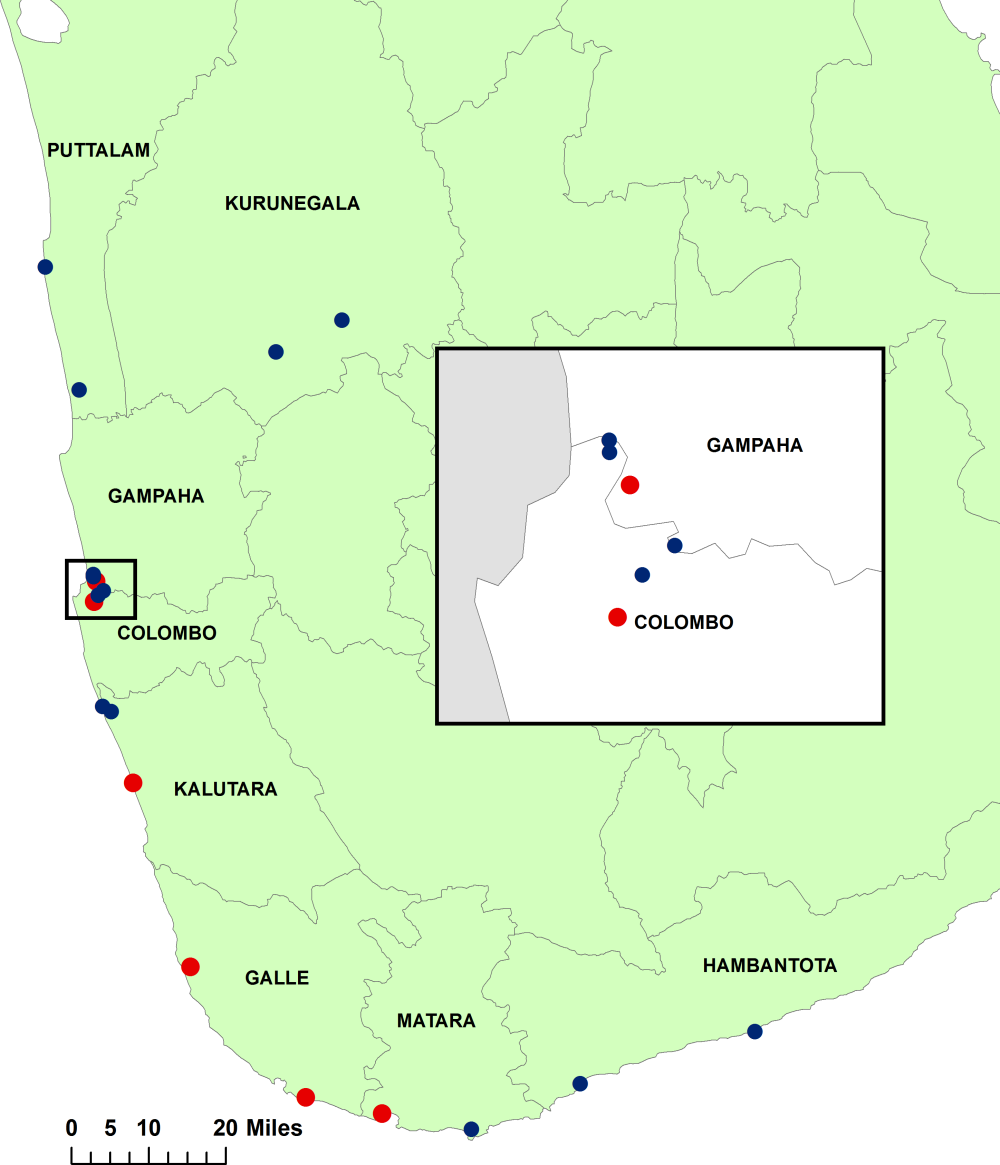
The map shows 8 filariasis endemic districts with approximate locations of 19 sentinel sites that were surveyed in 2011–2013 (blue and red circles). Six areas in red circles in 5 districts were reexamined in 2015–2017 for this study. The inset map shows surveyed sentinel sites in Colombo city and in the adjacent Colombo and Gampaha districts.

The study was performed in four Public Health Inspector (PHI) areas, one Public Health Field Officer (PHFO) area and one in Colombo municipality area that had evidence of persistent LF in a post-MDA surveillance study of 19 areas that was conducted in 2011–13 [7] (Fig 1). PHIs are sub-district health administrative units with populations in the range of 10,000–30,000 that are comprised of smaller units called Public Health Midwife (PHM) areas. The current study was performed three to four years after the last evaluation. No treatment for filariasis was provided in this interval in 4 of these PHIs. One round of MDA with DEC plus albendazole was provided in two of the PHIs (Unawatuna and Ambalangoda in Galle district) in 2014.

### Blood collection

Field procedures were the same as those previously described [7]. Briefly, field teams for collection of demographic information and blood samples consisted of a medical officer, a Public Health Inspector, a data entry operator, a phlebotomist, and one or two assistants. 1.5 mm x 2.0 mm, blue and 21 G x 1.8 mm pink single use contact-activated BD-microtainer lancets (Fisher scientific, Pittsburgh, PA) were used for blood collection in community and school surveys, respectively. Blood samples were collected during the day. Approximately 300 to 400 μl of blood was collected by finger prick from each study participant into an EDTA coated blood collection vial (Fisher Scientific). Preprinted barcode labels were used to link samples to participant records. Samples were transported to the central Antifilariasis Campaign (AFC) laboratory in Colombo in coolers. Plasma was separated by centrifugation from blood samples from school surveys and stored at -80 C for antibody testing.

### Survey methods in communities and schools

Survey methods were the same as previously described [7]. Briefly, area maps, census information (the number of houses and the number of households, number of schools, and the number of primary grade children) were obtained from census records, voter lists, and from school principals and administrators [17]. Community surveys sampled approximately 500 participants (ages 10–70 years) in approximately 125 households per PHI/PHFO area (range 127–172 HH). Most houses in the study area have 3 or 4 residents in this age range. Young children tested in school surveys were too young for inclusion in the community surveys (no overlap). Systematic sampling was used for household and mosquito sampling in each PHM within the PHI area. The number of houses/households needed for each community survey (125) was divided by number of PHMs in the PHI to get the number of houses to be sampled in each PHM. That number was divided by 4 to get the number of houses to be sampled in each quadrant in each PHM area. The sampling interval for houses was calculated by dividing the number of houses that were to be sampled in that PHM quadrant. Households from all quadrants in the PHM were enrolled. To maintain consistency in sampling and to obtain geographically dispersed samples, only 4 subjects ≥ 10 years were enrolled per household with equal preference for males and females. School surveys were performed in all schools that served the sentinel area. Finger prick blood was collected from primary grade school children (grades 1 and 2, age 6–8) and community participants for antigen and antibody testing.

### Filariasis testing for human subjects

Circulating filarial antigenemia (CFA) was detected in finger prick blood samples with a rapid format card test (BinaxNOW Filariasis, Alere Inc., Scarborough, ME) according to the manufacturer’s instructions. Cards were read visually at 10 minutes. Antigen testing was performed within 24 hr of blood collection.

IgG4 antibodies to recombinant filarial antigen Bm-14 in human plasma were detected by microplate ELISA (Filariasis CELISA, Cellabs Pty Ltd, Brookvale, NSW, Australia) as previously described [18]. Plasma samples were tested in a single well per sample and all positive and borderline tests with OD values >0.35 were retested on a different day to confirm their positivity. Samples with OD values consistently >0.35 were considered to be positive for antibodies to Bm14.

Persons with a positive filiarial antigen test had night blood testing to detect microfilaremia (Mf) as previously described. Briefly, finger prick blood collected between 9 pm and 12 midnight was used to prepare three-line blood smears (60 μl total volume of blood tested) that were dried, fixed, stained with Giemsa, and examined by microscopy for the presence of Mf. Each stained slide was read by a single experienced microscopist who recorded the absence or presence of Mf and Mf count.

### Detection of filarial DNA in mosquitoes

*Culex quinquefasciatus* were collected with CDC gravid traps (Model 1712, John W. Hock Company, Gainesville, FL) as previously described [7, 19]. Briefly, traps were placed outside houses in shaded areas in all quadrants of each PHM to ensure proportional sampling from all areas in each PHI. Trapped mosquitoes were sorted, dried at 95^0^ C for 1 hr., and placed in tubes for later molecular testing. Four pools of twenty fed, gravid, or semigravid female *Cx*. *quinquefasciatus* were tested from each of 50 trapping locations per PHI. Extraction of DNA from mosquitoes and detection of *W*. *bancrofti* DNA by qPCR were performed at the AFC central laboratory as previously described [20].

### Data collection and data management

Demographic information was collected and entered into BLU phones (BLU products, Miami, FL) using preloaded survey forms with LINKS data collection software https://www.linkssystem.org. Cell phones are equipped with global positioning system (GPS) capability, and GPS coordinates were captured at each surveyed house and mosquito trap location. Enrollment forms collected information on age, gender, consumption of antifilarial medications during the 2000–2006 MDA, bed net use last night, and clinical signs of lymphedema (all self-reported). Participant data and specimens were linked to laboratory test results with preprinted barcode labels. Deidentified, cleaned data were transferred into Microsoft Excel (Microsoft Corp., Redmond, WA) for analysis.

### Spatial analysis

Households included in population surveys and mosquito trapping sites were mapped using ArcGIS 10.2.1 (ESRI, Redlands, CA).

### Statistical methods

Chi-squared or Fisher’s exact tests were used to assess the significance of differences in filariasis parameters (prevalence of surveyed persons positive for antigenemia and antibody and percentages of mosquito pools that contained filarial DNA). Prevalence of filarial DNA in mosquitoes (maximum likelihood and 95% CI) were estimated using Poolscreen 2.02 software [21, 22]. Filarial DNA prevalence values in mosquitoes were considered to be significantly different if there was no overlap in the 95% CI values for the two samples. Correlations between human and mosquito infection parameters were assessed with the Spearman rank test. Graphs were produced with GraphPad Prism 7 software (La Jolla, CA).

## Results

### Community survey results

Six areas in 5 districts were resurveyed for *W*. *bancrofti* infection parameters between January 2015 and February 2017. LF surveillance periods for sample collections were in Peliyagodawatta (Oct., Nov., 2011 and Jan., Feb., 2015); Kalutara North (Sept., Oct., 2011 and Oct., 2015); Ambalangoda (Nov., Dec., 2011 and March, May 2015); Unawatuna (Nov., Dec., 2011 and July, Aug., 2015); Weligama (June, Sept., 2012 and Nov., Dec., 2015); Borella (April 2013 and Oct.,2016 to Feb., 2017). A total of 5350 people from the six sentinel sites participated in the study. This total included 3123 people in community surveys (ages 10–70, mean age 38 years, 42% males) and 2227 children (age 6–8) in school surveys. Details for enrollment in community surveys by sentinel site are provided in Table 1. Few filarial lymphedema cases (34 of 3123, 1.1%) were identified during the survey. Reported bed net use was moderate to high (range 49% to 70%) in all PHIs studied except Borella.

**Table 1:** Population and demographic information of community subjects enrolled in reexamination studies in selected sentinel sites.

District (IU)	PHI/PHFO	Area code	Population Size	Number of HH^c^	Number of HH surveyed (%)	Number of people Enrolled	Age (mean)	Male (%)	Bed net use (%)	Filarial Lymphedema (%)
Colombo	Borella ^a^	C4	137,423	27,484	127 (0.5)	508	35.3	41.4	24.0	0.4
Gampaha	PeliyagodaW ^b^	G3	10,560	2112	133 (6.3)	514	35.9	44.2	49.5	2.1
Kalutara	Kalutara North	KA2	11,728	2032	172 (8.5)	528	39.3	42.6	70.0	1.3
Galle	Ambalangoda	GL1	13,373	2792	152 (5.4)	523	39.9	40.1	64.4	1.3
	Unawatuna	GL2	16,636	3660	149 (4.1)	524	38.8	42.5	63.9	0.9
Matara	Weligama	M2	10,521	2104	169 (8.0)	526	37.0	40.3	67.8	0.6
			200,241	40,184	902 (2.2)	3,123	38	42	63	1.1

^a^ Sentinel site C4 is part of the Colombo Municipal Council area.

^b^ Sentinel site G3 is a public health field officer (PHFO) area in a semiurban zone adjacent to Colombo in Gampaha.

^c^ Approximate number of households (HH).

Survey results are summarized in Table 2. CFA prevalences were lower than 2% in all areas, but upper confidence limits for CFA were greater than 2% in the two PHI areas in Galle district. All CFA tests were negative in two PHIs. CFA prevalence was higher in males than in females in PHIs with at least one positive card test [13 of 869 (1.5%, 0.8–2.5 CI) vs. 3 of 1211 (0.2%, 0.1–0.7 CI), *P* = 0.003]. CFA prevalence was higher in adults (age ≥ 18) than in children (ages 10–17) in the community surveys [15 of 1731 (0.9%, 0.5–1.4 CI) vs. 1 of 349 (0.3%, 0.05–1.60 CI), *P* = 0.2]. Ten of 16 persons with positive CFA tests were over the age of 50 (range 54–69); eight of 10 (80%) CFA positives in this age group were males. Mf prevalences were well under 1% in all surveyed PHI areas. Three of 16 persons with positive CFA tests in the community surveys were also Mf positive (range 2–9 Mf count in 60 μl) with one each from Ambalandgoda, Unawatuna and Weligama.

**Table 2:** Summary of filariasis test results from community and school surveys.

District	PHI/PHFO	Area code	Mf Comm Positive/Total (%, 95% CI)	CFA Comm Positive/Total (%, 95% CI)	Mf School Positive/Total (%, 95% CI)	CFA School Positive/Total (%, 95% CI)	Ab School Positive/Total (%, 95% CI)
Colombo	Borella	C4	0/506 (0, 0–0.7)	0/506 (0, 0–0.7)	0/372 (0, 0–1.0)	0/372 (0, 0–1.0)	0/360 (0, 0–1.0)
Gampaha	PeliyagodaW	G3	0/512 (0, 0–0.7)	2/512 (0.4, 0.1–1.4)	0/366 (0, 0–1.0)	1/366 (0.3, 0.05–1.5)	2/335 (0.6, 0.1–2.1)
Kalutara	Kalutara North	KA2	0/528 (0, 0–0.7)	0/528 (0, 0–0.7)	0/380 (0, 0–1.0)	0/380 (0, 0–1.0)	9/378 (2.4, 1.3–4.5)
Galle	Ambalangoda	GL1	1/520 (0.2, 0.3–1.0)	5/520 (1.0, 0.4–2.2)	0/379 (0, 0–1.0)	1/379 (0.3, 0.0–1.5)	8/353 (2.3, 1.1–4.4)
	Unawatuna	GL2	1/523 (0.2, 0.0–1.0)	8/523 (1.5, 0.8–2.9)	1/359 (0.3, 0.0–1.5)	4/359 (1.1, 0.4–2.8)	14/333 (4.2, 2.5–7.0)
Matara	Weligama	M2	1/525 (0.2, 0.0–1.0)	1/525 (0.2, 0.0–1.0)	0/371 (0, 0–1.0)	0/371 (0, 0–1.0)	8/367 (2.2, 1.1–4.2)

PHI, Public Health Inspector area; Areas C4 and G3 are PHFO areas. Comm, community; CFA, circulating filarial antigen; Mf, microfilaremia; Ab, IgG_4_ antibody to Bm14 recombinant filarial antigen by ELISA.

### School survey results

CFA prevalence was low in all PHIs (Table 2), but the upper 95% CI exceeded 2% only in the Unawatuna PHI (Galle district). Only one of 5 CFA-positive children was Mf-positive (1 mf/60 μl). Antibody prevalences were higher in the 3 PHI areas in the Southern province (upper CI close to or higher than 5%) than in the other sentinel sites (Table 2). No child had a positive antibody test in Borella.

### Mosquito monitoring and molecular xenomonitoring

MX results are summarized in Table 3. Filarial DNA was detected in mosquitoes in all of six PHI areas. However, filarial DNA prevalence exceeded the target (upper CI > 1%) in three PHIs (Unawatuna and Ambalangoda in Galle district and Weligama in Matara district). Many trap locations were positive for mosquitoes with filarial DNA in Unawatuna, Ambalangoda and Weligama (Table 4). The percentages of positive mosquito trap sites in PHIs were not significantly correlated with percentages of houses with at least one CFA positive person (Table 4; Spearman rank correlation, r = 0.5, *P* = 0.2). Analysis on few data points in 6 areas may have caused this poor correlation.

**Table 3:** *Wuchereria bancrofti* DNA prevalence in *Culex quinquefasciatus* mosquitoes in survey areas.

District	PHI/PHFO	Area code	Survey Year	Number of mosquitoes tested	Number of pools tested	Number (%)^a^ of positive pools	*P* value	Filarial DNA prevalence^b^ in mosquitoes
Colombo	Borella	C4	2013	4000	200	26 (13)		0.69 (0.4–1.0)
			2016	4000	200	13 (6.5)	P = 0.043*	0.34 (0.2–0.6)
Gampaha	PeliyagodaW	G3	2011	4080	203	17 (8)		0.43 (0.2–0.7)
			2015	4000	200	9 (4.5)	P = 0.001*	0.23 (0.1–0.4)
Kalutara	Kalutara N	KA2	2011	4080	204	28 (14)		0.74 (0.4–1.0)
			2015	3986	200	10 (5)	P = 0.004*	0.26 (0.1–0.4)
Galle	Ambalangoda	GL1	2011	4000	200	52 (26)		1.40 (1.0–2.0)
			2015	3993	200	42 (21)	P = 0.28	1.17 (0.8–1.6)
	Unawatuna	GL2	2011	4000	200	54 (27)		1.50 (1.1–2.0)
			2015	4002	200	44 (22)	P = 0.29	1.23 (0.8–1.7)
Matara	Weligama	M2	2012	4080	204	51 (25)		1.40 (1.0–1.9)
			2015	4080	204	40 (19.6)	P = 0.23	1.09 (0.7–1.5)

^a^ % of total pools with mosquitoes positive for filarial DNA.

^b^ Filarial DNA prevalence (%,maximum likelihood and 95% CI) in mosquito pools were estimated using Poolscreen 2.02 software. *P* values shown are for differences relative to % pools positive in 2011, 2013 MX surveys in the same areas. % of pools positive for filarial DNA were significantly lower in Borella, Peliyagodawatta and Kalutara North*. *P* values are based on Chi-square analysis

**Table 4:** Filarial infections by household and by trap sites in 6 Public Health Inspector (PHI) areas.

PHI/PHFO	Number (%) of CFA positive houses	Number (%) positive mosquito trap sites
Borella	0/127 (0)	11/50 (22)
PeliyagodaW	2/133 (1.5)	7/50 (14)
Kalutara North	0/172 (0)	10/50 (20)
Ambalangoda	5/152 (3.3)	24/50 (48)
Unawatuna	7/149 (4.7)	26/50 (52)
Weligama	1/169 (0.6)	25/51 (49)

*W*. *bancrofti* infections were detected by antigen (CFA) testing. Filarial DNA was detected by qPCR with *Culex* collected in gravid traps.

### Spatial analysis of filarial infections in humans and mosquitoes

**Fig 2:**
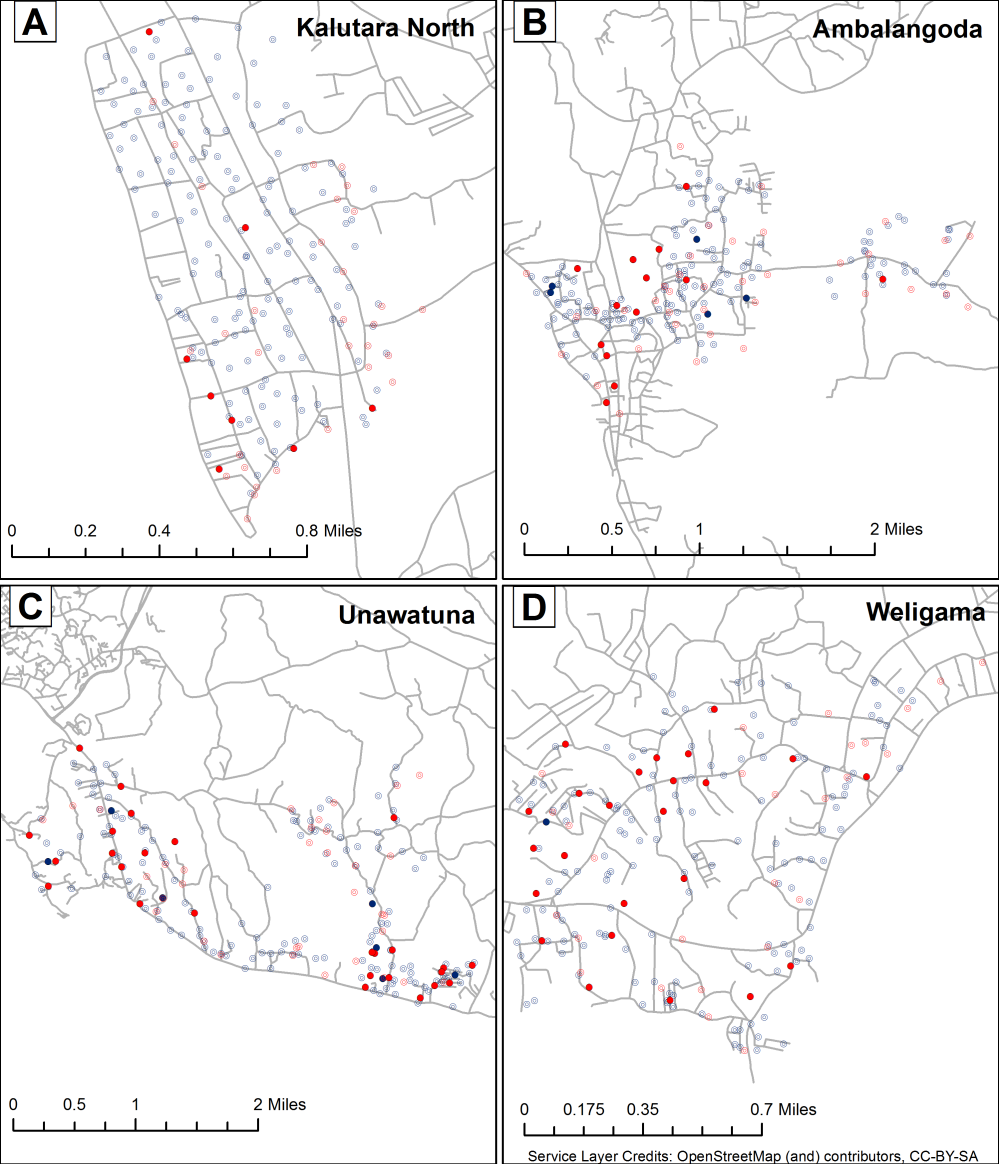
**Distribution of households and mosquito collection sites tested for filariasis in Kalutara North (A), Ambalangoda (B), Unawatuna (C) and Weligama (D) PHI areas.** Blue open circles indicate households (HH) where all tested residents had negative filarial antigen tests; Solid blue circles indicate houses with at least one resident with a positive filarial antigen test. Trap sites with no mosquito pools positive for filarial DNA are shown with open red circles, and trap sites with one or more positive mosquito pools are shown with solid red circles. Relatively few CFA positive households were identified, whereas there were many positive mosquito trap locations (especially in PHIs located in Galle and Matara districts).

The maps in Fig 2A–2D show locations for households surveyed for CFA and mosquito trapping sites in 4 PHIs. Many more positive mosquito trapping locations were identified than positive households. Positive mosquito trap sites were widely dispersed in three PHIs where approximately 50% of trap locations yielded mosquitoes with filarial DNA. In contrast, positive mosquito trap sites in the Kalutara North were concentrated in the southern part of that PHI.

### Community anti-filarial antibody prevalence in two PHI areas with persistent LF

**Fig 3:**
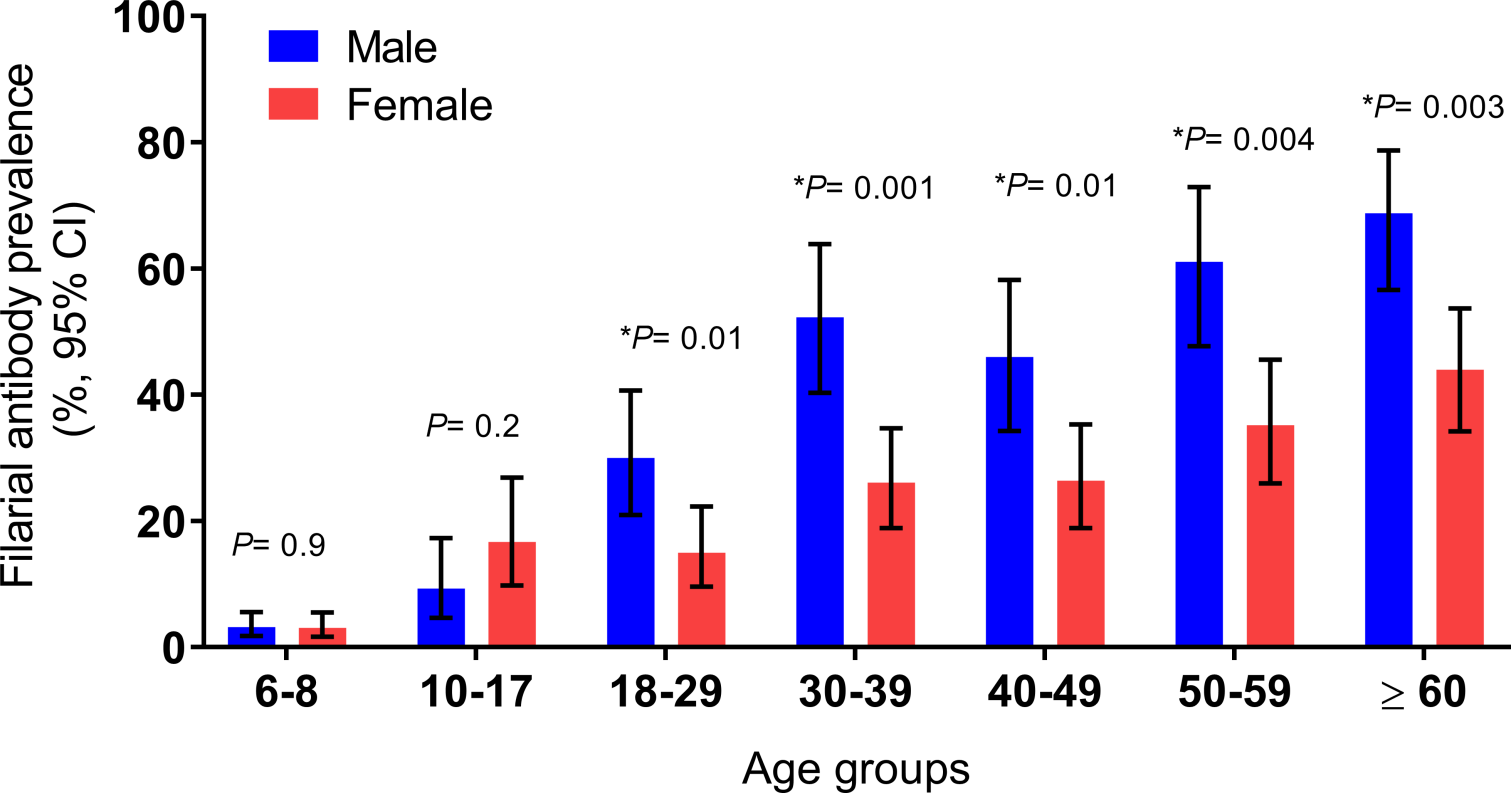
Summary of prevalence data for community and school antibody (Bm14) positives in 2 PHI areas Unawatuna (Galle district), Weligama (Matara district) in southern province. Data shown are antibody prevalence (%, 95% CI) by age and gender. Antibody prevalence in school children within these two communities are shown for 6–8 age for comparison. Antibodies to recombinant filarial antigen Bm14 were much more frequent in adults than in school age children (ages 10–17), and antibody prevalence was much higher in adult males than in females. Significance test results (*P values) on antibody prevalence in males are shown for each age group above the bars.

Community antibody testing was performed in Unawatuna and Weligama PHI areas that had evidence of persistent *W*. *bancrofti* infections and transmission in the baseline surveys. Community (age ≥10) antibody prevalence was very high in Unawatuna (168/506, 33%, 95% CI 29–37%) and in Weligama (166/501, 33.1%, 95% CI 29–37%), and these values were much higher in adults (age ≥18) than those in children (Fig 3). Only a small number of people with positive antibody tests were positive for CFA (6/168 in Unawatuna and 1/166 in Weligama). Overall anti-filarial antibody prevalence was significantly higher in males than females (42% vs 26%, *P* = 0.0001, combined results from Unwatuna and Weligama) (Fig 3).

### Comparison of *W*. *bancrofti* infection parameters over time in 6 PHI areas

Results from surveys conducted in Peliayagodawatta in 2008, 2011 and 2015 are summarized in Table 5. *W*. *bancrofti* infection parameters spontaneously improved over time in this area, and most of these changes were statistically significant. In addition, the trend was consistent over time with greater reductions from baseline 2008 values in 2015 than in 2011.

**Table 5:** Summary of *Wuchereria bancrofti* infection parameters in Peliyagodawatta in 2008, 2011 and 2015.

LF infection markers	# tested 2008	Prevalence	# tested	Prevalence	# tested	Prevalence
		% (95% CI)	2011	% (95% CI)	2015	% (95% CI)
		2008		2011		2015
Mf Community	944	0.4 (0.16–1.0)	504	0.4 (0.1–1.4) (NS)	512	0 (0–0.7) (NS)
CFA Community	945	3.8 (2.7–5.2)	504	1.2 (0.5–2.5)	512	0.4 (0.1–1.4)
				(*P* = 0.004)*		(*P* = 0.0001)*
CFA age 6–8	265	1.9 (0.8–4.3)	377	0.3 (0.05–1.5)	366	0.3 (0.05–1.5)
				(*P* = 0.03)		(*P* = 0.03)
Bm14 Ab (6–8)	ND	ND	350	4.3 (2.6–6.9)	335	0.6 (0.1–1.5)
						(*P* = 0.03)*
Filarial DNA prevalence in mosquitoes. MLE^a^ (% 95% CI)	277 Pools	0.75 (0.52–1.06)	204 Pools	0.43 (0.24–0.71)	200 Pools	0.23 (0.10–0.45)
% pools positive for filarial DNA^b^		39/277	17/204 (8.3%, 5.3–13)		9/200 (4.5%, 2.4–8.3)	
		(14%,10.5–18.7)	*P* = 0.06*		*P* = 0.001*	

^a^ MLE: Maximum Likelihood Estimates by PoolScreen in %.

^b^ Percentages of total number of pools with mosquitoes positive for filarial DNA. *P* values are based on Chi-square. NS, Not significant, ND, Not done. *P* values shown in 2011 results column are for differences relative to results of baseline studies performed in 2008. *P* values shown in 2015 results column are for differences compared to results of baseline studies in 2008 for antigenemia in community and school, microfilaremia in school children, and filarial DNA in mosquitoes. *P* values were calculated for differences in antibody prevalence in school children between 2011 and 2015. *P* values with “*” symbol were statistically significant.

**Fig 4:**
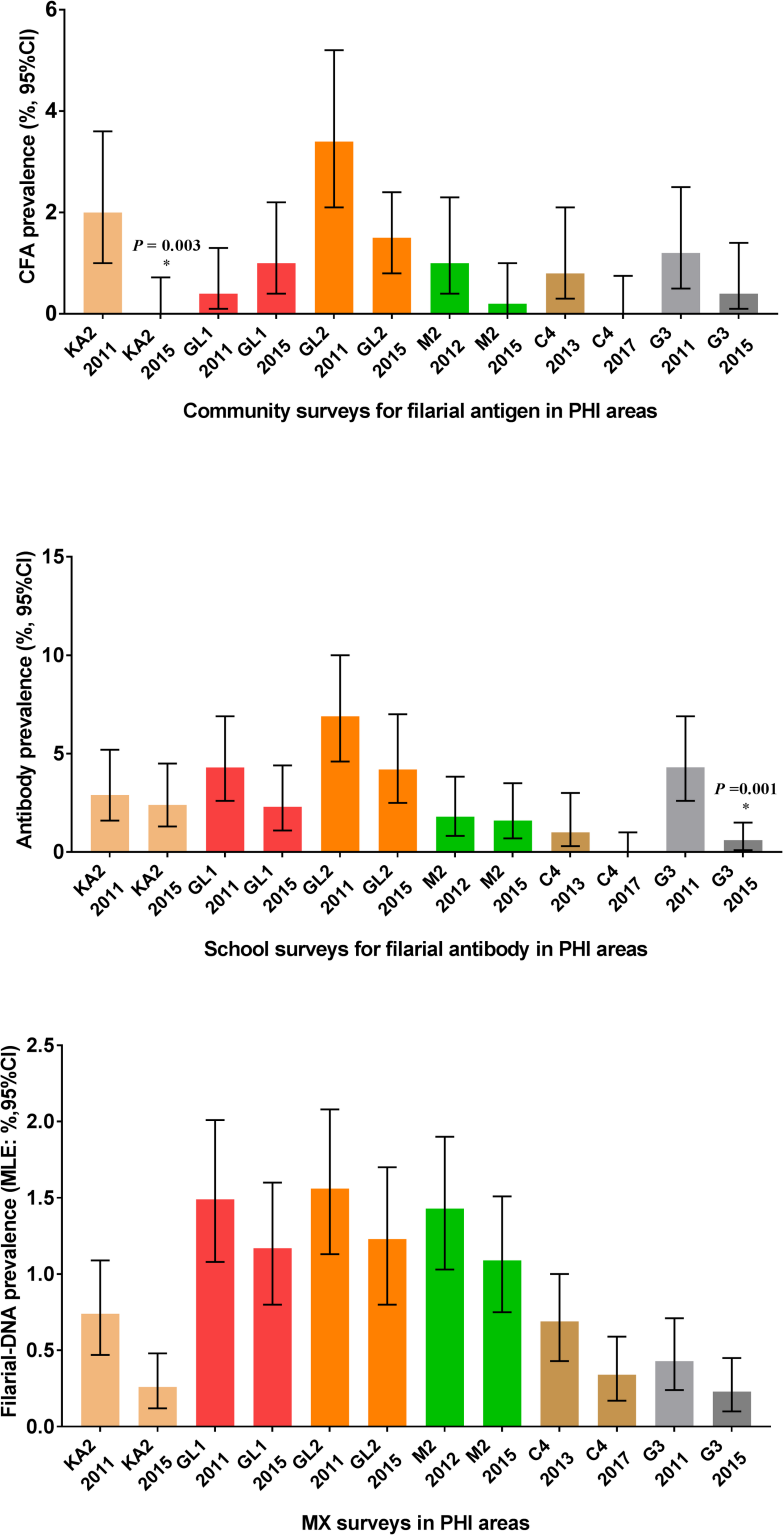
Comparison of comprehensive filariasis surveillance data for Kalutara North (KA2), Ambalangoda (GL1), Unawatuna (GL2), Weligama (M2) and Borella (C4) and Peliyagodawatta (G3) sentinel sites in Sri Lanka. Data shown are prevalence for LF parameters in community and school children and prevalence of filarial DNA in mosquitoes (% with 95% confidence intervals). Significance results with P* values shown for KA2, and G3 are for differences in prevalence for these LF parameters between years in the same sites.

Longitudinal data on LF infection parameters from all six PHIs are shown in Fig 4. CFA and MX results improved significantly between 2011 and 2015 in Peliyagodawatta and Kalutara North and between 2013 and 2017 in Borella. There was also a slight downward trend in some LF parameters in Unawatuna, Ambalangoda and Weligama, but these changes were not statistically significant even though the government provided one round of MDA with DEC plus albendazole in Unawatuna and Ambalangoda during this interval. Filarial DNA prevalences in mosquitoes were high (MLE > 0.25%, upper CI > 1%) in 2015 in these PHIs.

### Negative association of bed net use with filarial infection prevalence

CFA prevalence was significantly lower in Unawatuna and Ambalangoda community participants (n = 1043, age ≥10) who reported bed net use (0.60% *vs* 2.42% in non-users, *P* = 0.01). There was no association between anti-filarial antibody and bed net use when all ages were considered in Unawatuna and Weligama, but antibody prevalence in children aged 10–17 were significantly lower in bed net users than in nonusers (8/96, 8.3% vs.12/62, 19.4%, *P* = 0.04).

## Discussion

This study has provided interesting new data on changes in LF parameters post-MDA. LF elimination requires reduction of infection parameters to levels that cannot sustainably support transmission. This does not mean that all measures of LF must be zero, and indeed we found evidence of low-level persistence of LF in all 6 PHI areas that were restudied in 2015–16. Breakpoints for LF transmission (where filariasis parameters have been reduced below levels required for sustained transmission) are poorly defined, and they depend on many factors that are difficult to measure and may vary widely between and within endemic regions. We have proposed targets for LF elimination programs [7, 23], and the current study attempted to “ground truth” these targets. While the time intervals and number of study sites were not sufficient to rigorously prove the validity of the targets, our results suggest that they are in the right range and also feasible for measurement by national programs. Results from Sri Lanka are likely to apply to many other LF-endemic areas with transmission by *Culex* mosquitoes. Long-term post-MDA surveillance will be needed to verify LF elimination in areas like Sri Lanka that have highly competent vectors.

As in our prior study, data from 2015–16 again show that MX and antibody testing of school children are more sensitive than antigen testing of school children for detecting low-level persistence of LF in post-MDA settings. Results for 3 parameters measured in this study and in 2011–13 (community CFA, antibody prevalence in school children, and prevalence of filarial DNA in mosquitoes) support the provisional target values for LF elimination programs (upper 95% CI values of 2% for community CFA, 5% for antibody in school children, and 1% for MX), and areas that failed to meet one of the targets often failed to meet the others. Results from Peliyagodawatta suggest that the community CFA target of 2% in the post-MDA setting may be too conservative, because the prevalence in that study site declined from 3.8 in 2008 to 0.4% in 2015 without intervention.

One weakness of school-based TAS as currently performed is that signals from focal high infection areas are often diluted when evaluation units are large. Evaluation Units with populations of one million or more are commonly employed by LF elimination programs in Asia [1, 4]. The ideal EU size is not known, but reducing the population for EUs to 200,000 or lower should be more sensitive for detecting persistence or resurgence of LF than the currently recommended ceiling of 2 million. Large EUs were needed to reduce surveillance costs when the cost of CFA tests was high. However, recent changes have reduced these costs, and this may make it feasible to reduce EU size and perform more TAS. A recent study has modeled effects of EU size and population on sensitivity for detecting ongoing hotspots of transmission [24]. It is not clear whether this information can be translated into changes in policy or practice that are feasible for use by national LF elimination programs.

While school-based TAS with a point of care antigen test is a convenient way to sample a sentinel population for recent infections, this approach is less sensitive than the other parameters that we tested. The strategy of sampling sentinel populations does not work well if the sentinels are at low risk for infection. Antigen data in this study and in our prior study show that adult males have much higher filarial infection prevalence than other groups in Sri Lanka, and they represented the bulk (>80%) of the residual reservoir of infection in PHI areas surveyed in this study. Is this because they have more exposure to infective mosquitoes, higher susceptibility to infection, and lower compliance with MDA, or a combination of these factors? A TAS that focuses on high-risk adult males to assess the persistent reservoir of infection might be a more effective tool for post-MDA surveillance than school-based TAS that aims to detect recent infections.

Antifilarial antibody test results (reflecting both recent and past filarial infections) also showed age-related increases in prevalence, and antibody prevalence was much higher in males than females. These results underscore gender and age differences in LF infection and exposure in Sri Lanka. A post-MDA surveillance study in American Samoa found similar results with increased infection prevalence in adult males [25]. Our finding of high antibody prevalence in adults in areas that were close to LF elimination suggest that testing adults with this antibody test (IgG4 antibodies to recombinant filarial antigen Bm14) has little value as a post-MDA surveillance tool. Forty-one of 1,700 (2.3%) children tested in 5 PHI areas with one or more child with a positive antibody test had positive antibody tests, and 6 of these children had positive antigen tests. These children were born after Sri Lanka’s national MDA program was completed. Prior studies have shown that persons with positive antifilarial antibody tests have an increased risk for developing microfilaremia during follow-up [26, 27], so we recommend presumptive treatment for children with positive antibody tests. AFC currently provides antifilarial treatment to persons with microfilaremia or positive antigen tests according to WHO guidelines.

The longitudinal results in this study are especially interesting. They suggest that filariasis parameters in Peliyagodawatta in 2008 and in Kalutara North and Borella in 2011–13 were already below transmission breakpoints, and LF appears to be on a glide path to elimination in these areas. On the other hand, results from Unawatuna, Ambalangoda, and Weligama suggest that transmission is ongoing in these areas and that they will require further intervention. Of the various parameters measured, the filarial DNA prevelance in mosquitoes (as assessed by MX) seems to have been the best predictor for LF persistence. Positive MX results in areas where little or no infection was detected in humans (Borella, Kalutara North, and Peliyagodawatta) is intriguing. It is likely that there are infected persons in these communities who were non-compliant with MDA in the past and also not sampled in our community surveys. Mosquitoes do not ask permission when they conduct night blood sampling, and this probably accounts for the enhanced sensitivity of MX for detecting persistent infections relative to other modalities.

Results from this study provide useful insights regarding approaches for clearing up LF transmission hotspots in post-MDA settings like those in southern Sri Lanka. Resumption of MDA is not an efficient option when human infection prevalence is very low; MDA will not benefit the vast majority of people who are uninfected, and the program is likely to miss most persons with persistent infection who have been non-compliant with MDA in the past. The Sri Lanka AFC provided one round of MDA with DEC plus albendazole in 2014 (prior to this study), and also provided MDA to selected areas within Galle district in 2015 and 2016. We believe that instead of focusing on the percentage of the population that can be reached with MDA (population coverage), programs should focus on how to optimize treatment of infected persons (worm coverage). Since more than 80% of those with antigenemia in the present study and approximately 65% of those with antigenemia in the study published in 2014 were adult males, a “test and treat” program or other approaches that focus on adult males might result in higher worm treatment coverage than population-based MDA. Population antigen data and antibody data from children in Unawatuna and Weligama PHIs in Galle and Matara districts point to a potential protective effect of bed nets for *Culex*-transmitted LF in Sri Lanka. This was an unexpected finding, because bed nets are considered to be more important for LF control in settings with anopheline transmission [28]. Bed nets are popular in Sri Lanka, because they help to reduce the mosquito nuisance and because they may provide some protection against dengue virus infection. Additional promotion of bed nets or a focused government subsidy program for bed nets in selected areas with persistent LF may help to clear remaining LF hotspots.
